# Transcutaneous supraorbital neurostimulation in “de novo” patients with migraine without aura: the first Italian experience

**DOI:** 10.1186/s10194-015-0551-3

**Published:** 2015-07-22

**Authors:** Antonio Russo, Alessandro Tessitore, Francesca Conte, Laura Marcuccio, Alfonso Giordano, Gioacchino Tedeschi

**Affiliations:** Headache Center, Department of Medical, Surgical, Neurological, Metabolic and Aging Sciences, Second University of Naples, Naples, 80138 Italy; MRI Research Center SUN-FISM, Second University of Naples, Naples, Italy; Institute for Diagnosis and Care “Hermitage Capodimonte”, Naples, Italy

**Keywords:** Migraine, Transcutaneous supraorbital neurostimulation, tSNS, Therapy, Cefaly

## Abstract

**Background:**

Transcutaneous supraorbital neurostimulation (tSNS) has been recently found superior to sham stimulation for episodic migraine prevention in a randomized trial. We evaluated both the safety and efficacy of a brief period of tSNS in a group of patients with migraine without aura (MwoA).

**Methods:**

We enrolled 24 consecutive patients with MwoA experiencing a low frequency of attacks, which had never taken migraine preventive drugs in the course of their life. Patients performed a high frequency tSNS and were considered “compliant” if they used the tSNS for ≥ 2/3 of the total time expected. For this reason, four patients were excluded from the final statistical analysis. Primary outcome measures were the reduction migraine attacks and migraine days per month (p < 0.05). Furthermore, we evaluated the percentage of patients having at least 50 % reduction of monthly migraine attacks and migraine days. Secondary outcome measures were the reduction of headache severity during migraine attacks and HIT-6 (Headache Impact Test) rating as well as in monthly intake of rescue medication (p < 0.05). Finally, compliance and satisfaction to treatment and potential adverse effects related to tSNS have been evaluated.

**Results:**

Between run-in and second month of tSNS treatment, both primary and secondary endpoints were met. Indeed, we observed a statistically significant decrease in the frequency of migraine attacks (*p* < 0.001) and migraine days (*p* < 0.001) per month. We also demonstrated at least 50 % reduction of monthly migraine attacks and migraine days in respectively 81 and 75 % of patients. Furthermore, a statistically significant reduction in average of pain intensity during migraine attacks (*p* = 0.002) and HIT-6 rating (*p* < 0.001) and intake of rescue medication (*p* < 0.001) has been shown. All patients showed good compliance levels and no relevant adverse events.

**Conclusion:**

In patients experiencing a low frequency of attacks, significant improvements in multiple migraine severity parameters were observed following a brief period of high frequency tSNS. Therefore, tSNS may be considered a valid option for the preventive treatment of migraine attacks in patients who cannot or are not willing to take daily medications, or in whom low migraine frequency and/or intensity would not require pharmacological preventive therapies.

## Background

Migraine is a common neurological disorder characterized by episodes of unilateral or bilateral headache lasting for hours to days, which may be accompanied by photophobia, phonophobia, nausea and vomiting. It is well-known that a primary brain dysfunction leads to episodic activation and sensitization of the trigeminovascular pain pathway during attacks. However, a functional and anatomic relationship exists between peripheral afferent nerves supplying the head and neck and the brainstem, subcortical and higher order brain processing centers [1]. Pharmacological anti-migraine preventive therapies are widely used to reduce the impact of migraine on quality of life; nevertheless, they may exhibit incomplete efficacy and significant side effects [2]. On the other hand, there is some evidence that interventions targeting peripheral nerves are able to modulate neuronal circuits involved in pain control and that they could be useful in some selected patients with migraine [3].

In the last years, percutaneous neurostimulation, by means of nerve fibres depolarization produced by electrical impulses from a current generator device, has been applied in patients with migraine [4]. However, this approach is burdened by surgical invasive procedures to implant the electrodes and the neurostimulator and it has been used only in the most severe migraine conditions [5–7]. More recently, supraorbital neurostimulation (tSNS) of the upper branches of the trigeminal nerves was found superior to sham stimulation for episodic migraine prevention in a previous randomized trial in a large cohort of patients with migraine [8]. Moreover, this technique has the advantage of being non-invasive, safe and almost devoid of adverse effect [9]. Therefore, tSNS treatment might be a valid option for the prophylactic treatment in patients who cannot take daily medications or in those experiencing a low migraine frequency and/or intensity when pharmacological preventive therapies may be not strictly indicated [10].

Therefore, the aim of the present study was to specifically evaluate the efficacy and tolerability of tSNS in this patients population. To this end, we used a tSNS medical device, called Cefaly® (CEFALY Technology, Herstal, Belgium), approved to be used for migraine prevention by the Food and Drug Administration (FDA) and for sale in Europe. To best of our knowledge, at the moment, this study represents the first Italian experience of tSNS in patients with migraine.

## Methods

### Patient population

According to the International Headache Society (IHS) criteria of International Classification of Headache Disorders [11] and to the more recent ICHD-3 beta version criteria for MwoA [12] twenty-four consecutive patients with MwoA experiencing a frequency of attacks ≤ 5/month, were prospectively recruited from the migraine population referring to the outpatient headache clinic of the First Neurological Clinic at the Second University of Naples. All patients had normal neurological examination. Exclusion criteria were the presence of any other type of headache, somatic or psychiatric conditions, and intake of daily medication. To avoid any possible pharmacologically related interferences on primary and secondary endpoints, the patients were drug-naïve for any pharmacological anti-migraine preventive therapies (*i.e.,* they had never taken anti-migraine preventive drugs in the course of their life). Demographic data and the following clinical features were obtained from the patients: disease duration, migraine attacks per month and frequency (day/month), average of pain intensity during migraine attacks (by means of visual analogic scale - VAS) and intake of rescue medication during migraine attacks. All patients completed the HIT-6 (Headache Impact Test) (see Table 1 for demographic and clinical features). Triptans and NSAID (including acetaminophen) were taken by patients for rescue treatment. No patient was taking combination analgesics for migraine attacks. Patients were not allowed to use the tSNS as rescue treatment for migraine attacks. All patients underwent preliminary MRI examination before entering the present study, to exclude any relevant brain structural abnormality.

**Table 1:** Clinical features of patients with MwoA

Parameter	Timing	Mean ± SE	*p* value
Gender		15 F/5 M	
Age (years)		32.9 ± 2.3	
Disease duration (years)		8.3 ± 1.7	
Frequency (days/month)	Baseline	4.5 ± 0.24	< 0.001
	Follow-up	2.06 ± 0.28	
NSAID intake (including acetaminophen)	Baseline	3.2 ± 0.6	0.02
	Follow-up	1.3 ± 0.4	
Triptans intake	Baseline	2.4 ± 0.7	0.04
	Follow-up	0.9 ± 0.3	
Total intake of rescue medication	Baseline	5.6 ± 0.4	< 0.001
	Follow-up	2.2 ± 0.3	
HIT-6	Baseline	62.3 ± 1.4	< 0.001
	Follow-up	53.1 ± 1.4	
VAS of attack intensity	Baseline	8.0 ± 0.1	0.002
	Follow-up	6.7 ± 0.2	

*F* female, *M* male, *NSAID* non-steroidal anti-inflammatory drugs, *HIT-6* Headache Impact Test, *VAS* Visual Analogue Scale

### Neurostimulation protocol

Consistent with previous studies [8, 9], tSNS was delivered with a 30 mm × 94 mm self-adhesive electrode placed on the forehead and covering the supratrochlear and supraorbital nerves bilaterally. The tSNS generates biphasic rectangular impulses with an electrical mean equal to zero and with following characteristics frequency: 60 Hz, pulse width: 250 μs and intensity: 16 mA. The tSNS sessions lasted twenty minutes/day.

### Standard protocol approvals, registrations, and patient consents

The experiments conformed to the principles of the Declaration of Helsinki and were approved by the ethics committee of the Second University of Naples. All participants provided informed, written consent after the study procedure had been explained.

### Study design

The study was conducted from January 2013 to October 2014. Patients' baseline clinical features were determined using data from the 28-day baseline diaries. The baseline day was followed by a 60-days treatment period without intermediate visits and a final evaluation at the end of the tSNS protocol. Patients filled in diaries recording migraine occurrence and its severity on a 10-points scale (0: no pain; 10: severe pain prohibiting daily activities) and intake of rescue medication during migraine attacks. A migraine day was defined as a day with headache fulfilling ICHD-III (beta version) [12] criteria for MwoA, except for duration, if the attack was treated. Migraine days not separated by at least one headache-free day were considered to belong to the same migraine attack. At the end of 60-days tSNS treatment, during the final visit, the Italian version of the HIT-6 questionnaire was filled in by patients [13]. Finally, compliance and satisfaction to treatment and potential adverse effects (AE) related to tSNS have been also evaluated. Compliance to treatment was assessed by a built-in electronic system allowed recording usage of the stimulators by each patient. Although a previous study considered that an inclusive treatment of 400 min is necessary and sufficient to obtain a therapeutic effect from tSNS [9], patients were considered “compliant” and included in the analysis if they used the Cefaly® device for ≥ 800 min during the 60-days of treatment (≥ 2/3 of the total time expected). For this reason, four patients were excluded from the final statistical analysis. Patients were considered “satisfied” if they expressed the desire to continue the tSNS treatment. Patients were asked if they had experienced AE, according to previous described AE in patients using Cefaly® device [9].

### Outcome measures

Primary outcome measures were: a) significant change in migraine attacks per month; b) significant change in migraine days per month. Furthermore, we also evaluated the patients with MwoA as percentage of responders (*i.e.,* percentage of patients having at least 50 % reduction of monthly migraine attacks and migraine days) between run-in and 60-days of treatment. Secondary outcome measures were: a) significant reduced average of pain intensity during migraine attacks; b) significant reduced HIT-6 questionnaire rate; c) reduced intake of rescue medication during migraine attacks per month. Finally, we assessed the percentage of: a) patients that are satisfied or not satisfied with the treatment; b) patients showing compliance to the treatment; c) patients experiencing AE from the treatment.

### Statistical analysis

Statistical analysis was performed with STATA software, version 13 (STATA Corp., Texas, USA). Continuous data were expressed as the mean ± standard error (SE) and compared using the paired *t*-test and Wilcoxon matched-pairs signed-rank test where appropriate. Changes in clinical parameters were expressed in percentage variation. For all analyses, statistical significance was defined as *p* < 0.05.

## Results

**Fig. 1:**
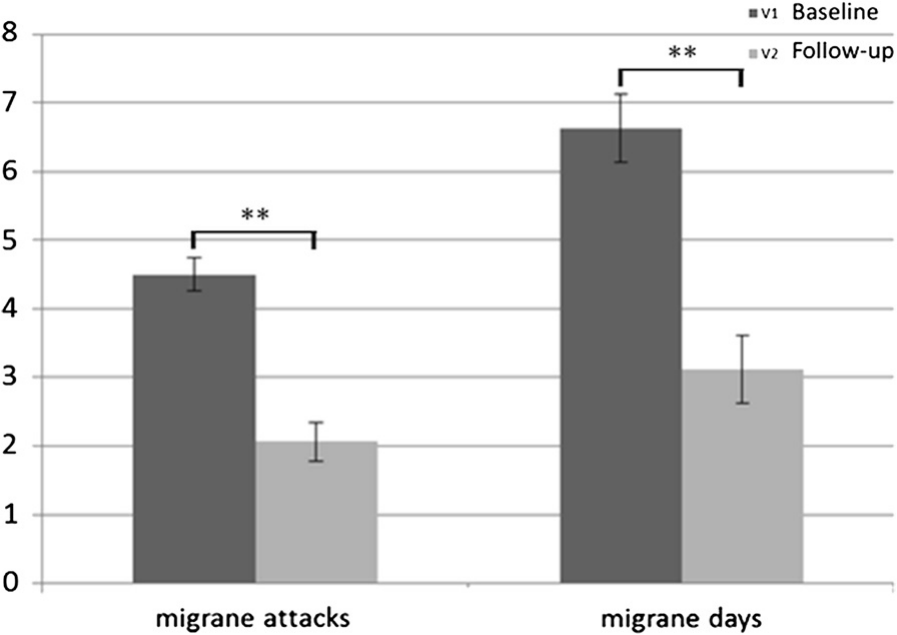
Significant differences in migraine attacks (*p* < 0.001) and migraine days (*p* < 0.001) between run-in and 60-days of treatment

**Fig. 2:**
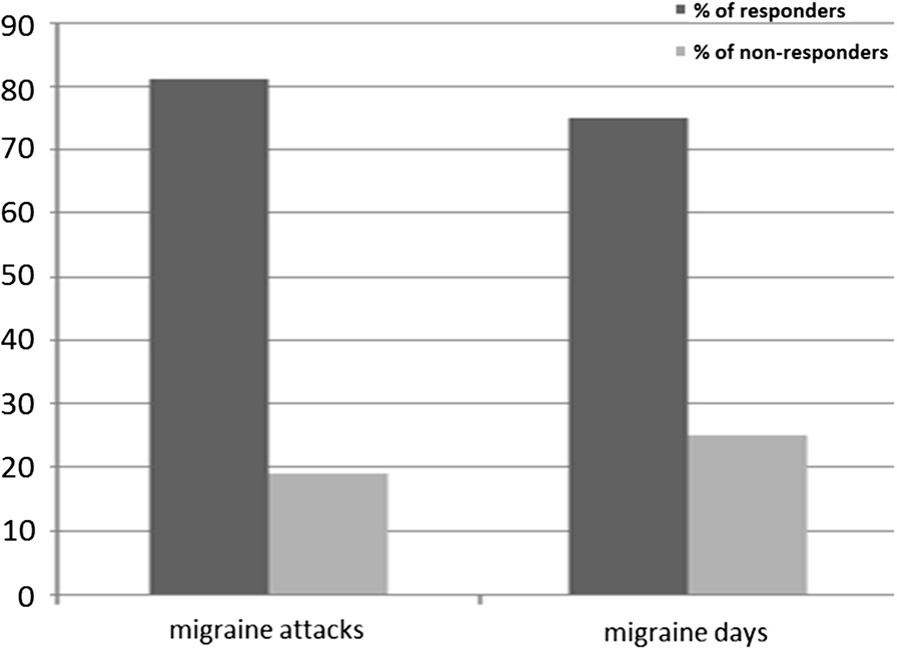
Percentage of patients having at least 50 % reduction of monthly migraine attacks and migraine days between run-in and 60-days of treatment

Based on patients’ diaries, between baseline and at the end of 60-days tSNS treatment, all primary and secondary endpoints were both met. Indeed, we observed a statistically significant decrease in the frequency of migraine attacks (*p* < 0.001) and migraine days per month (*p* < 0.001) (see Fig. 1). Considering primary outpoints as percentage of responders, we demonstrated at least 50 % reduction of monthly migraine attacks and migraine days in respectively 81 and 75 % of patients, between run-in and 60-days of treatment (see Fig. 2).

**Fig. 3:**
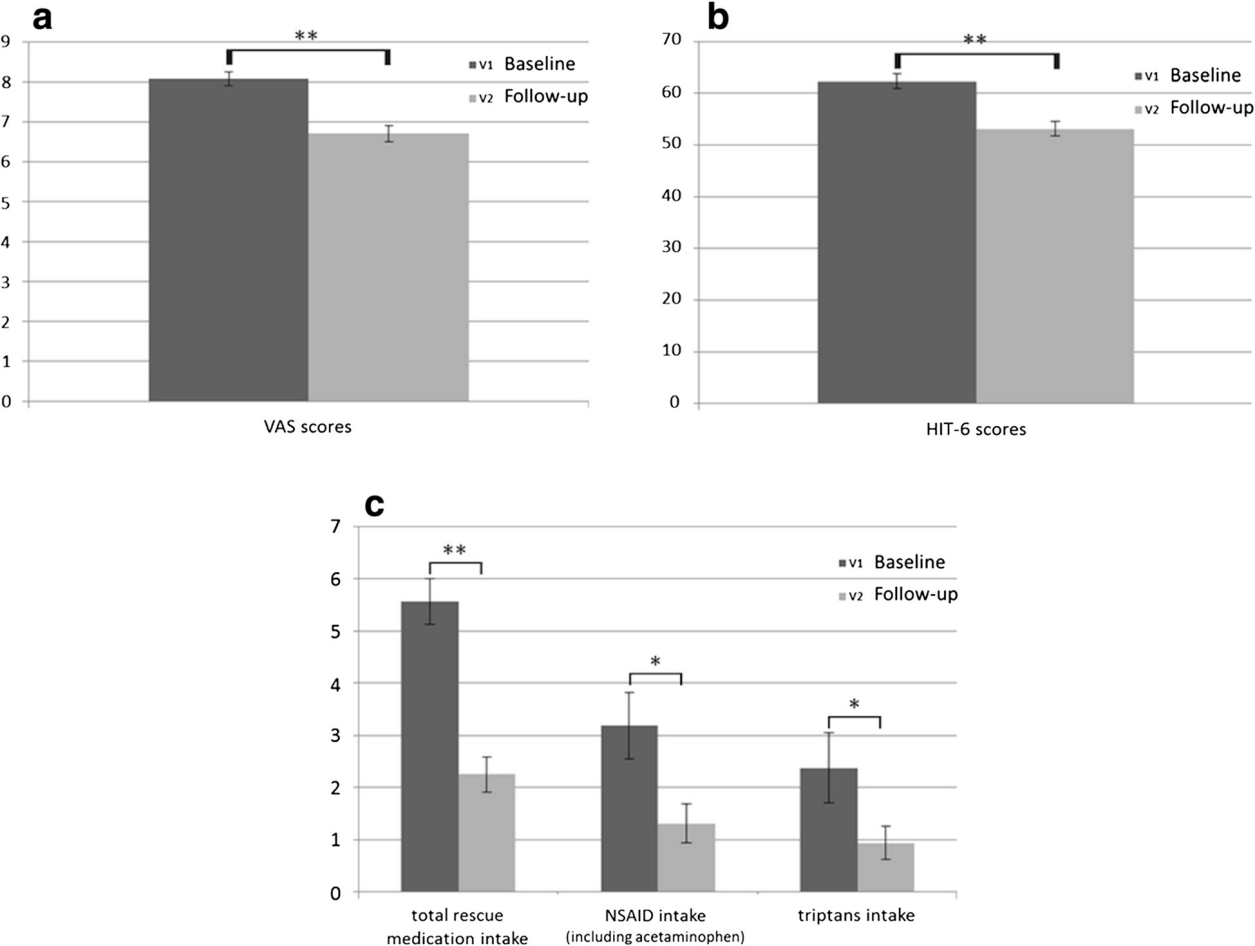
Significant reduction in **a** mean headache severity during migraine attacks (*p* = 0.002); **b** HIT-6 questionnaire rating (*p* < 0.001) and **c** monthly intake of total rescue medication intake (*p* < 0.001), NSAID (including acetaminophen) (*p* = 0.02), and triptans intake (*p* = 0.04)

Furthermore, a reduced average of pain intensity during migraine attacks (*p* = 0.002) (see Fig. 3a) and HIT-6 rating (*p* < 0.001) (see Fig. 3b) has been observed at the end of the treatment. A reduced intake of rescue medication during migraine attacks per month (*p* < 0.001) has also been noticed. A sub-analysis showed a statistically significant reduction in the intake of rescue medication, both non-steroidal anti-inflammatory drugs (NSAID) (including acetaminophen) (*p* = 0.02) and triptans (*p* = 0.04), during the 60-days tSNS treatment (see Fig. 3c).

All patients (20/20 patients) showed no adverse events during the tSNS period and were willing to continue the treatment at the end of the protocol.

## Discussion

Taken together, our results provide evidence that daily treatment with tSNS has a preventive effect in patients with MwoA experiencing a low frequency of attacks and drug-naïve for any pharmacological anti-migraine preventive therapies. This preventive effect has been achieved after a relatively short-term (*i.e.,* 60-days) tSNS treatment. Furthermore, the tSNS seems to be devoid of AE and patients with MwoA exhibit a high level of both compliance and satisfaction with the treatment.

The goals of the pharmacological anti-migraine preventive therapies include reduction of migraine attack frequency, intensity, duration and disability; improvement of health-related quality of life and avoiding of migraine attacks escalation and medication misuse [14]. However, migraine patients could reasonably decide not to use pharmacological anti-migraine preventive therapies for different reasons such as negative attitudes towards medication in general and fear of medication side-effects [15, 16]. Among these latter, the increase of weight (especially in female patients) and/or sedation seems to be the most important factors [16, 17]. Unfortunately, in the last decade, there have been no new migraine preventive drugs, with a good level of efficacy and safety, that can be used in clinical practice.

In this context, neurostimulation has inaugurated a new era in headache management and offers an alternative to pharmacological therapy. Neurostimulation treatments were first experimented in patients with refractory or intractable headaches, then they have been applied as an alternative to acute or preventive therapies when pharmacological strategies produce unsatisfactory effects or are associated to unacceptable AE [2]. Recently, a 3-month treatment with tSNS was estimated superior to sham stimulation for episodic migraine prevention in a randomized trial in a large cohort of patients [8]. Moreover, although a reduced vigilance in healthy volunteers due to tSNS treatment was evidenced [18], tSNS was considered a safe and well-tolerated headache treatment, providing a high rate of satisfaction in patients with migraine which used the device for a 40-day period [9].

By comparison, tSNS appears not to match the preventive benefits seen, for example, with topiramate, a first choice drug in migraine preventive treatment. Indeed, topiramate can decrease the number of migraine days by 44 % as opposed to a 25 % reduction of days using tSNS, and the number of migraine attacks in the course of topiramate treatment was reduced by 48 %, while the tSNS reduced the number of migraine attacks by 19 % [19, 20]. Nevertheless, we believe there is a need to further evaluate whether tSNS could exhibit a more robust efficacy in specific migraine patients sub-groups or clinical phenotypes.

Our data demonstrate that preventive treatment with tSNS may have a strong benefit in a selected population of patients with MwoA, characterized by low migraine frequency. We believe that these patients could be more prone to successfully respond to tSNS due to a minor impact of migraine burden on pain pathways. Indeed, converging evidence suggest that repeated migraine attacks are related to functional and structural changes induced by dural nociceptors stimulations [21]. Specifically, a high frequency of migraine attacks may be related to trigemino-spinal central sensitization and impaired descending pain inhibitory controls [22]. Moreover, the above-mentioned pathophysiological mechanisms seem to be associated with an atypical stimulus-induced activation of brainstem, subcortical, and cortical regions involved in sensory processing both during and between migraine attacks [22]. Finally, tSNS could be more efficacious in those patients where the pain processing network has not been already influenced by previous pharmacological preventive therapies. It is a common experience that some migraine medications work better in patients drug-naïve for any pharmacological anti-migraine therapies, compared with those with a past marred by previous preventive treatments. Indeed, it is well-known that pharmacological anti-migraine preventive therapies, including antiepileptic drugs, beta-blockers, tricyclic antidepressants and calcium-channel blockers, tend to modify activities in both central and peripheral nervous system [23].

Our findings are in line with previous data of tSNS efficacy in patients with migraine [8]. Interestingly, in our patients both migraine attacks frequency and migraine days per month showed a percentage of at least 50 % of reduction, that is usually considered clinically relevant [24], in respectively 81 and 75 % of patients, between run-in and 60-days of treatment.

Furthermore, the remaining attacks exhibited a significant reduction in both the average of pain intensity and their impact on patients’ quality of life.

As a consequence of the significant improvement of migraine burden in patients with MwoA, a reduced total intake of rescue medications has been noticed during the 60-days tSNS treatment. Specifically, a significant reduction has been observed in both NSAID (including acetaminophen) and triptans intake in our patients.

Our findings are also in line with previous data of compliance, safety and satisfaction with tSNS treatment in patients with migraine [8, 9]. All patients with MwoA showed a very high compliance levels to treatment and no AE occurred during the tSNS period. Finally, all patients reported that they were willing to continue tSNS treatment even after the end of the study.

We are aware that the present study is not exempt from some limitations. First of all, we did not use a tSNS sham device and, therefore, we cannot rule-out the possible role of a placebo-effect on primary and secondary outcomes in our study. In particular, several factors may contribute to the remedial efficacy of tSNS in our patients such as alternative form of medical therapy, patients naïve to preventive treatment and observation period limited to no more than two months. However, the placebo-effect seems to have a lower impact in the prophylactic treatment than in the acute treatment of migraine attacks. This could be due to the inherent variability in response measured over a period of months compared with one measured over a period of hours [25]. Moreover, the effective tSNS superiority respect to sham stimulation for the prevention of migraine headaches has been extensively demonstrated in a previous randomised controlled trial in a large cohort of patients with migraine [8]. Nevertheless, in partial disagreement with our findings, Schoenen and colleagues [8] did not show statistically significant effect on migraine attacks at two months, although ameliorating effect on migraine severity vanished in sham treated patients and amplified in effectively treated patients at this time of the study. We suggest that a greater migraine severity (*i.e.,* frequency of migraine per month and disease duration) and, probably, previous pharmacological anti-migraine preventive therapies may cause a different impact on pain pathways in the two migraine populations and consequent different response to the tSNS treatment. Second, the lack of blinding may weaken the results of the present study. However, empirical evidence shows that although double-blind randomized controlled trials are the gold standard for proving efficacy of a therapeutic procedure, they often suffer from lack of generalizability [26]. Therefore, we believe that our data, in addition to the previous effectiveness and safety results of double-blind randomized controlled studies [8], could provide additional information which may be useful in everyday clinical practice [26]. Finally, although our results are consistent with previous studies [8, 9], our sample size was relatively small. Therefore, further studies are needed to corroborate our findings and to explore tSNS efficacy and tolerability in patients with migraine compared with preventive treatments used in clinical practice.

## Conclusion

In conclusion, we can confirm that our findings do not differ very much from previous observations [8, 9], but rather extend them. Indeed, we believe that tSNS could be considered a first choice therapy in selected migraine populations due to the high level of efficacy and safety. Therefore, tSNS treatment could be a valid option for the prophylactic treatment of migraine attacks in patients who cannot or are not willing to take daily medications, or in which migraine frequency and intensity did not allow clinicians to fully consider or offer pharmacological anti-migraine preventive therapies to their patients [10].
